# Approaches to passive mosquito surveillance in the EU

**DOI:** 10.1186/s13071-014-0604-5

**Published:** 2015-01-08

**Authors:** Helge Kampen, Jolyon M Medlock, Alexander GC Vaux, Constantianus JM Koenraadt, Arnold JH van Vliet, Frederic Bartumeus, Aitana Oltra, Carla A Sousa, Sébastien Chouin, Doreen Werner

**Affiliations:** Institute of Infectology, Friedrich-Loeffler-Institut, Federal Research Institute for Animal Health, Suedufer 10, 17493 Greifswald– Insel Riems, Germany; Public Health England, Porton Down, Salisbury, UK; Wageningen University, Wageningen, The Netherlands; ICREA Movement Ecology Laboratory (CEAB-CSIC), Girona, Spain; Instituto de Higiene e Medicina Tropical, Universidade Nova de Lisboa, Lisbon, Portugal; EID Atlantique, Rochefort-sur-Mer, France; Institute for Land Use Systems, Leibniz Centre for Agricultural Landscape Research, Muencheberg, Germany

**Keywords:** Passive surveillance, Community participation, Citizen science, Invasive mosquitoes, Vectors, Mosquito inventory

## Abstract

The recent emergence in Europe of invasive mosquitoes and mosquito-borne disease associated with both invasive and native mosquito species has prompted intensified mosquito vector research in most European countries. Central to the efforts are mosquito monitoring and surveillance activities in order to assess the current species occurrence, distribution and, when possible, abundance, in order to permit the early detection of invasive species and the spread of competent vectors. As active mosquito collection, e.g. by trapping adults, dipping preimaginal developmental stages or ovitrapping, is usually cost-, time- and labour-intensive and can cover only small parts of a country, passive data collection approaches are gradually being integrated into monitoring programmes. Thus, scientists in several EU member states have recently initiated programmes for mosquito data collection and analysis that make use of sources other than targeted mosquito collection. While some of them extract mosquito distribution data from zoological databases established in other contexts, community-based approaches built upon the recognition, reporting, collection and submission of mosquito specimens by citizens are becoming more and more popular and increasingly support scientific research. Based on such reports and submissions, new populations, extended or new distribution areas and temporal activity patterns of invasive and native mosquito species were found. In all cases, extensive media work and communication with the participating individuals or groups was fundamental for success. The presented projects demonstrate that passive approaches are powerful tools to survey the mosquito fauna in order to supplement active mosquito surveillance strategies and render them more focused. Their ability to continuously produce biological data permits the early recognition of changes in the mosquito fauna that may have an impact on biting nuisance and the risk of pathogen transmission associated with mosquitoes. International coordination to explore synergies and increase efficiency of passive surveillance programmes across borders needs to be established.

## Background

During the past few years, Europe has become increasingly affected by invasive mosquitoes and mosquito-borne disease cases/outbreaks [1,2]. The Asian tiger mosquito *Aedes albopictus* (*Stegomyia albopicta* sensu Reinert et al. [3]), the Asian bush mosquito *Ochlerotatus japonicus japonicus* (*Hulecoeteomyia japonica japonica* sensu Reinert et al. [4]) and the yellow fever mosquito *Aedes aegypti* (*Stegomyia aegypti* sensu Reinert et al. [3]) have recently established or re-emerged in parts of Europe and started spreading [5-7]. While *Ae. albopictus* and *Ae. aegypti* are known to be efficient vectors in the field [8,9] and were responsible for historic and recent epidemics/cases of disease in Europe and European overseas territories [e.g. 10-19], *Oc. j. japonicus* has not yet been confirmed to be a vector in the field but has proven vector competence for several viruses in the laboratory [7].

In addition to invasive mosquito species, there are a number of native species capable of transmitting pathogens such as viruses [20,21], malarial parasites [22] or filarial worms [23] which are constantly transported internationally/intercontinentally and introduced due to the ever-growing mass transportation of animals and humans [24].

Alerted by these recent developments, various European countries have started mosquito monitoring and surveillance programmes, in part combined with a screening of the collected mosquitoes for pathogens [25]. Usually traps are deployed for determining the occurrence and the spatiotemporal distribution of the culicids. However, managing a network of traps that covers a whole country is not only expensive but also extremely time- and labour-consuming. In addition, not all mosquito species are attracted to the commonly used trap systems and some may even remain unnoticed.

To assist active mosquito monitoring by trapping, some EU countries have launched passive surveillance activities, thereby using other data sources, such as existing databases, or addressing the general public. Such approaches provide plenty of additional data with minimum effort and high cost/benefit efficiency.

Incorporating the observations by the interested public in data collection, also known as citizen science, has become increasingly popular [e.g. 26,27]. Citizen science projects are of special relevance in mosquito research, as the presence of a nuisance species (native or invasive) is usually perceived for the first time by local inhabitants [e.g. 28,29]. Establishing efficient channels of communication between the community, scientists and authorities may therefore contribute to the early detection of changes in the mosquito fauna.

Projects from six European countries using passive strategies for mosquito surveillance, including community-based approaches, are presented together with some of their major outcomes. Challenges, drawbacks and future opportunities for intensified passive surveillance at the European scale are discussed.

### Germany: The “Mückenatlas” (mosquito atlas)

The “Mückenatlas” was launched in April 2012 as part of a German nation-wide mosquito monitoring programme run by the German Federal Institute for Animal Health (Friedrich-Loeffler-Institut) and the Leibniz Centre for Agricultural Landscape Research. In this project, citizens are requested to collect mosquitoes in their private surroundings, kill them and submit them to the research institutions involved. The mosquitoes are required to be captured undamaged while resting by putting any kind of closable container over them and to be put in the freezer overnight. They are then posted in a small non-breakable container with a completed questionnaire. The questionnaire which is downloadable from the project’s homepage (www.mueckenatlas.de) requests information on collection site and date, general weather conditions at the time of collection and a short description of the area where the mosquito was found. Optionally, the collectors can ask for their name or a synonym to be entered in an interactive mosquito collection site map presented on the homepage. The homepage also provides background information on the monitoring programme and on mosquitoes in general. In the long-term, it is intended to present mosquito distribution maps.

The mosquitoes submitted to the “Mückenatlas” are identified in the laboratory, either morphologically or, in the case of cryptic species and damaged specimens, genetically, and are added to a pinned voucher specimen collection and/or to a DNA collection. The results are fed into the German mosquito database CULBASE where all German research groups currently involved in mosquito field work enter their data. On request, the CULBASE data will be made available to scientists, stakeholders and policy makers.

Every participant to the “Mückenatlas” will personally be informed via email or ordinary mail on the identification result of her/his mosquito/es and usually be provided with some information on the biology of this particular species in order to develop a better understanding for hematophagous insects. Occasionally, advice on prevention and personal protection is given.

In order to draw public attention to the “Mückenatlas” and enhance public interest in mosquito research, considerable public relations work is done. Regularly, press releases are published, TV, radio and newspaper interviews given, articles contributed to magazines and flyers distributed.

**Figure 1:**
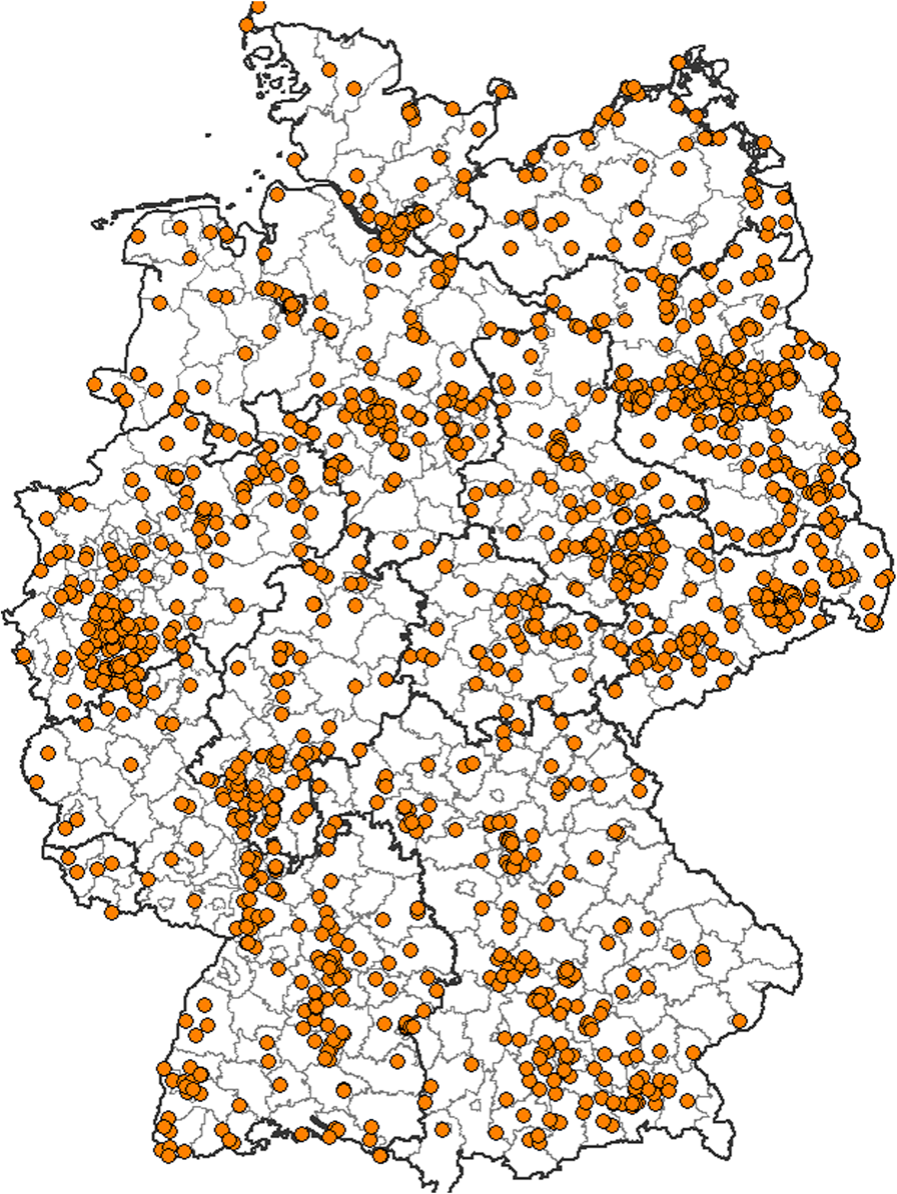
**Geographic distribution of German “Mückenatlas” mosquito collection sites 2013.**

In 2012, 2,020 postal items containing 6,127 mosquito specimens from 1,564 collection sites were submitted to the “Mückenatlas”. These numbers increased in 2013 to 2,440 postal items with 11,447 mosquito specimens from 1,864 sites. A geographically concentrated participation of the public leading to an agglomeration of collection sites in some German regions (Figure 1) is probably attributed to imbalanced media presence and metropolitan areas with higher human population densities.

In the first year, about 23% of the submissions did not contain mosquitoes but other arthropods such as spiders, beetles, grasshoppers, bugs and other dipterans. Although the error rate *per se* remained more or less the same over time (24% in 2013), the systematic relatedness of the submitted arthropods to the family Culicidae, i.e. the number of dipterans, gradually increased. Thus, the extensive media work probably had some educational effect on the public. This impression is supported by the fact that a lot of people contributing to the “Mückenatlas” sent mosquitoes repeatedly.

Several interesting and even surprising results emerged from the “Mückenatlas”. First, 39 of 50 mosquito species described for Germany were recorded in comparison to 36 species as collected within the monitoring programme by traps. Second, particularly rare species, such as *Culiseta glaphyroptera, Cs. ochroptera* and *Cs. alaskaensis*, were re-discovered after decades without record [30]. Third and most unexpectedly, two new populations of *Oc. j. japonicus* were detected in western and northern Germany, respectively [31,32]. The events leading to this detection will be briefly depicted. In early August 2012, five persons from the greater Bonn area in western Germany (federal state of North Rhine-Westphalia) independently submitted seven *Oc. j. japonicus* specimens to the “Mückenatlas”. Excluding coincidence, the area was instantly visited and scrutinized for *Oc. j. japonicus* larvae. These were quickly confirmed in the gardens or the immediate neighbourhoods of the senders’ homes and, subsequently, in plenty of cemeteries in an area covering approximately 2,000 km^2^. With their numerous flower vases and plant dishes, cemeteries both offer a lot of breeding places for mosquitoes and can be relatively quickly and efficiently checked [33]. Later in the same year, a single *Oc. j. japonicus* female was received from far further north in Germany, the metropolitan area of Hanover (federal state of Lower Saxony). The end of the mosquito season approaching, a site inspection was not carried out before May 2013. This time, only the central water reservoirs of cemeteries were checked. Again, larvae were detected over a huge area of about 500 km^2^. Probably rather than the West German population, which was only some 150 km in a direct line from the formerly known Belgian site of *Oc. j. japonicus* occurrence, would the northern German population have gone unnoticed if not for the “Mückenatlas”. Neither personal nor financial resources of the monitoring project would have justified a closer examination of northern German areas as a spread to that region was simply not considered.

Just recently (mid-August and mid-October 2014), the first two *Ae. albopictus* individuals were submitted to the “Mückenatlas”, which led to the detection of a local population breeding in southern Germany in late summer/autumn 2014 [34].

While the number of mosquitoes collected per site (one to a few specimens) is considerably lower in the “Mückenatlas” surveillance scheme as compared to the number obtained by traps, many more sites are considered. The larger geographic coverage leads to a better account of the distribution of many indigenous species and to a higher probability of early random findings indicating new developments in the indigenous mosquito fauna such as invasion by foreign species.

The success of the “Mückenatlas” is attributed to the eye-level dialogue between citizens (voluntary mosquito submitters) and scientists. It is a citizen science project with the highest possible level of data quality since the citizens do not convey non-verifiable observations, but make the observed objects available to the scientists who do the quality management (i.e. the identification) themselves.

### UK: Mosquito Reporting Scheme/Mosquito Watch

The Mosquito Recording Scheme (MRS) was set up by Public Health England (PHE, then the Health Protection Agency, HPA) and the Biological Records Centre (www.brc.ac.uk) in 2005. In the same way as with other species groups, the scheme would provide a national focus for Culicidae data in the UK, and the data would be made publicly accessible via the National Biodiversity Network Gateway (http://www.nbn.org.uk). The MRS built upon a previous mosquito database held by the University of East London which led to distribution maps of the British mosquitoes [35-40]. In addition to this founding data resource, the MRS receives datasets from amateur and professional entomologists, museums and universities, and also provides an identification resource for the general public to submit mosquitoes that may be causing a biting nuisance. The samples are sent to PHE for identification by medical entomologists who respond with information about the species and their habitats.

**Figure 2:**
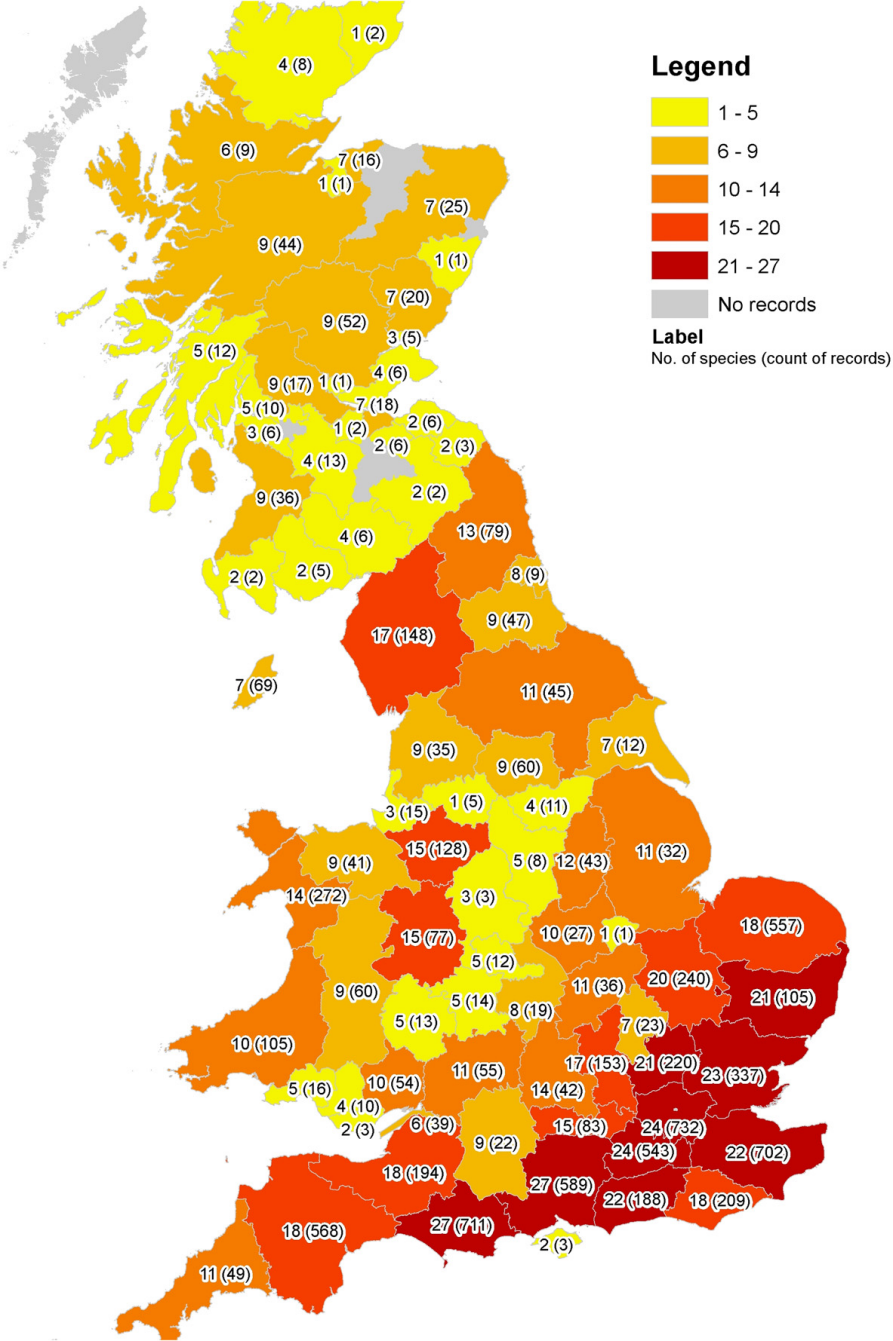
**Number of species per UK county (England and Wales) and lieutenancy area (Scotland).** The total number of records are shown in brackets.

Since 2005, the MRS has received approximately 3,500 submissions in addition to 7,000 records from historical datasets dating back to the 1750s. Whilst there are records for most counties across Great Britain, the majority of records come from the south-eastern and southern counties of England (Figure 2). Thirty-four different mosquito species have been recorded through MRS. The most common species are from the *Anopheles maculipennis* and the *Culex pipiens* complexes while the dataset also holds records of some very rare British species such as *Anopheles algeriensis, Aedes vexans, Ochlerotatus leucomelas, Ochlerotatus sticticus*, *Orthopodomyia pulcripalpis* and *Culex modestus*.

In 2005, the Medical Entomology group of HPA established a ‘Mosquito Watch’ scheme with the Chartered Institute of Environmental Health (CIEH) and Killgerm Ltd. to provide a forum for environmental health officers to submit mosquitoes for identification. From 2005 to 2012, there were 116 submissions, the majority of which were identified as *Cs. annulata* and *Cx. pipiens* s.l., the latter being hibernating females [40]. This scheme provides information on nuisance reports at a local authority level, but also acts as a forum for detecting invasive species. Indeed, several of the *Cs. annulata* reports were initially presented in the press as *Ae. albopictus*. The Mosquito Watch scheme now reports jointly with the MRS.

As a follow up to Mosquito Watch, the HPA, in collaboration with CIEH, conducted a questionnaire-based survey of local authorities in 2009 on mosquito nuisance [41]. This repeated previous surveys conducted in the 1960s, 1980s and 1990s by Service [42] and Snow [43,44]. In 2009, a total of 221 local authority pest control units supplied information on mosquito nuisance and control (64% response rate), with 57 (25%) indicating mosquito nuisance biting in the last ten years and 29 (13.7%) in the last 12 months. Eleven local authorities reported having conducted mosquito control within the last ten years with issues associated with *Oc. detritus, Cs. annulata, Oc. cantans* and *Cx. pipiens* s.l. [41]. This survey was able to confirm ongoing and persistent mosquito nuisance caused by *Oc. detritus* in salt-marsh areas in the Dee estuary (Cheshire) and at Sandwich (Kent), and nuisance biting by *Cx. pipiens* biotype *molestus* at sewage treatment sites in London. All sites continue to be subjected to mosquito control.

The records sent in to the MRS have contributed to the understanding of the distribution of mosquito species in the UK, and, based upon these records, 14 sites across the country were actively sampled in 2010 with the aim of studying the seasonality and abundance of the majority of British mosquito species. This study was initiated to provide contemporary data, rather than purely rely on historical records. It led to the discovery of *Cx. modestus* in North Kent [45,46] and of new locations of rare species, such as *Oc. leucomelas* (Haverthwaite, Cumbria), *Ae. vexans* (Sandwich, Kent) and *Oc. sticticus* (Hurcott, Worcestershire), and confirmed the persistence of restricted species, such as *An. algeriensis* (Hickling, Norfolk).

The MRS and Mosquito Watch are important and affordable tools that provide a medical entomology resource for the UK. They enable better responses to nuisance biting issues, an early warning system for invasive mosquitoes and provide a repository for records collected by a range of people which can be shared with the public, pest controllers, government officials and academics.

### The Netherlands: The “Muggenradar” (mosquito radar)

The “Muggenradar” (www.muggenradar.nl) is a surveillance instrument initially launched to investigate mosquito activity during winter. To obtain information on perceived nuisance by mosquitoes during wintertime (itch as a result of a bite or mosquito buzzing during sleep), the Dutch general public was addressed in January 2014 by a specific call for reporting mosquito activity. The duration of the call was intended to be five weeks. It was accompanied by a press release and the establishment of a website. The press release contained information on the rationale and goals of the call and instructions on how to access the website and submit observations and mosquito specimens. The website included a mapping functionality, general background information on the biology of mosquitoes, the possibility of establishing contact by social media and, most importantly, an online questionnaire. Via the questionnaire, the participants provided information on whether or not they experienced nuisance, the type of nuisance experienced, the location where the nuisance occurred and the date. Participants were given the possibility to submit a mosquito specimen for further identification. The website also had a link to contact the responsible scientists via email.

Besides the website, a Facebook (www.facebook.com/muggenradar) and a Twitter (@muggenradar) account were launched. Through these social media regular updates on the status of the project were provided. After the press release, various radio and TV agencies covered an item on the call and reported on the numerous mosquito submissions arriving at the laboratory.

Although it was initially hard to gauge whether people would be interested in filling out the questionnaire, catching mosquitoes and submitting them, the launch was a large success as measured by the number of participating households and the constructive and positive feedback.

**Figure 3:**
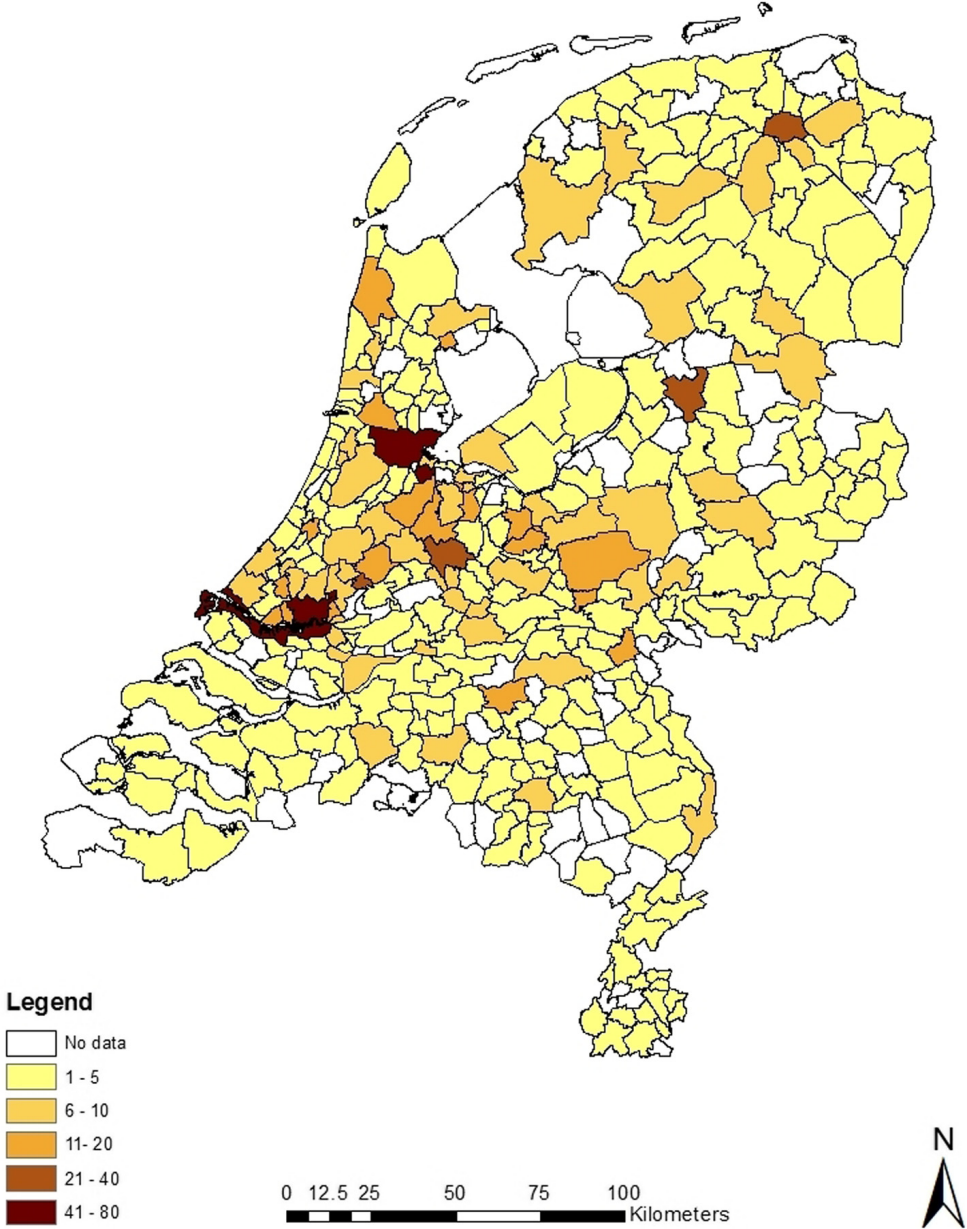
**Number of submitted mosquito samples per Dutch municipality in the framework of the five-week , “Muggenradar” call in January and early February 2014. The two ‘41-80 sample’ municipalities are Amsterdam (in the north) and Rotterdam (in the south).** The four ‘21-40 sample’ municipalities are the three provincial capitals Groningen, Zwolle and Utrecht (from north to south) and Gouda.

In total, 3,624 online questionnaires were filled out within the defined five-week period. Of the 2,724 (75%) submitted samples, 1,563 (57%) were mosquitoes while the remainder did not belong to the Culicidae, but to other dipteran families (e.g. winter crane flies) or groups of insects. The relative distribution of submitted mosquitoes in The Netherlands is shown in Figure 3. The map reflects highly populated areas, but spatial statistical analyses are ongoing to detect whether there are true hotspots of mosquito nuisance, independent of human population density. Of all submitted samples, only 128 (5%) were beyond recognition upon arrival in the laboratory. Of the Culicidae, 930 (60%) were of the genus *Culex*, while the others were *Culiseta* (34%) or *Anopheles* (7%). No sample was received that pointed in the direction of a non-native mosquito species.

In 312 of the received envelopes (11%), more than one mosquito was submitted. In 112 cases (4.1%), traces of blood were seen in the mosquito, indicating that they had recently fed.

At this stage, genetic analyses on the mosquitoes are still ongoing. The identification results will eventually produce a map of mosquito presence and nuisance during the winter and provide more insight into the ecology of mosquito species.

The positive performance of the “Muggenradar” can probably be attributed to several aspects: (i) clear packaging and posting instructions supported by pictures on the website were provided, ensuring that the mosquito specimens arrived in a relatively good state for morphological and genetic identification; (ii) postage costs were covered (envelopes could be sent to a response number that ensures payment by the receiver), thereby lowering the threshold to submit mosquitoes, and (iii) completing the questionnaire would take only a few minutes as the requested information was strictly limited to what was considered essential.

Although it was stressed that submitted samples would not be screened for the presence of pathogens, it was communicated that the obtained information is highly valuable for assessing transmission risks of mosquito-borne pathogens. Thus, information on the potential role of mosquitoes in the spread of diseases was included in the communication. In only a few cases, concerns from the public related to potential contraction of disease were received, and these were all answered individually.

By no doubt, the personalized feedback to every single participant, including information on whether a culicid (in that case which genus), a non-culicid or a non-identifiable specimen was submitted, was an important element of the “Muggenradar”. In many cases, this feedback was again replied to by positive emails.

In conclusion, this first community-based project for collecting information about mosquito activity and biting nuisance during winter in The Netherlands was very successful although it is realized that at this stage nothing can be said about the relative size of the nuisance that was experienced. Rather than having a continuous call, two calls per year that are opened for a relatively short period of time each (e.g. two weeks in winter and two weeks in summer) are considered preferable in the future since this approach will limit the burden of administrative and taxonomic work, while still achieving sufficient mosquito samples for reliable population estimates.

### Spain: AtrapaelTigre.com (hunting the tiger)

“AtrapaelTigre.com” started in 2013 as a pilot project, aiming at exploring alternatives to traditional and costly environmental awareness actions for *Ae. albopictus* in Catalonia, northeastern Spain. Since its first detection in 2004 near Barcelona [28], *Ae. albopictus* has spread southwards along the Spanish Mediterranean coast. Detection patterns suggest a spread in jumps, with *Ae. albopictus* detected quickly in locations distant from the initial sightings [47-52]. Currently, the abundance of *Ae. albopictus* is very high in some urban areas. In Catalonia, for example, the species requires considerable direct control and management costs and non-negligible indirect costs to the touristic and real-estate sectors [53]. Due to the high direct costs, surveillance and control efforts are mainly restricted to specific locations and regions at certain times.

“AtrapaelTigre.com” is led by a research group on movement ecology (ICREA Movement Ecology Laboratory, CEAB-CSIC), funded primarily by FECYT (Spanish Foundation for Science and Technology) and supported by an increasing number of other public and private institutions. The project builds upon three main pillars: i) face to face training workshops, ii) a multi-purpose online space (i.e. the project website, www.atrapaeltigre.com) and iii) a mobile phone app (Tigatrapp), the main participatory element. Using the app, citizens are asked to report adult tiger mosquito sightings and breeding spots that are automatically updated on a map on the project website. For this, volunteers answer a survey consisting of three questions about the mosquito/breeding site characteristics used for data validation purposes, add the location coordinates using either GPS or selecting a location on a map, and may also voluntarily attach pictures, write accompanying notes and send possible tiger mosquito specimens by post.

The pilot project was initially targeted at approx. 6,000 primary school students that participated, through their schools, to a tiger mosquito educational programme in the province of Girona (Catalonia). It was strongly believed that before promoting the project to the entire country, a bounded approach was needed to test for the best solutions, engagement elements and quality assurance. With the help of schools, children were to involve parents, promoting a viral-communication effect to their families, causing the whole family unit to collect data during the summer. Each school received a participation guide and a password for app download through the project website. However, it was complicated to massively engage schools in a virtual environment in the short-term and during the summer, especially with novel technologies and involving very young students. Therefore, other citizens were also allowed to participate after sending a notification of interest and a password request. For this pilot project, dissemination was limited to the region of Catalonia only.

At the end of the pilot, 138 citizens (mostly regular citizens) with Android smartphones downloaded the app and 44 actively sent data. It was estimated that each technological barrier (e.g. app only available for Android, participation request by email, password needed for download, key needed for app activation) reduced the number of participants by around 50% at each step. As a recall, the initial purpose was not to have many participants, but raise awareness among kids at schools (and hence their families) and assess the web-app system with a few subjects.

**Figure 4:**
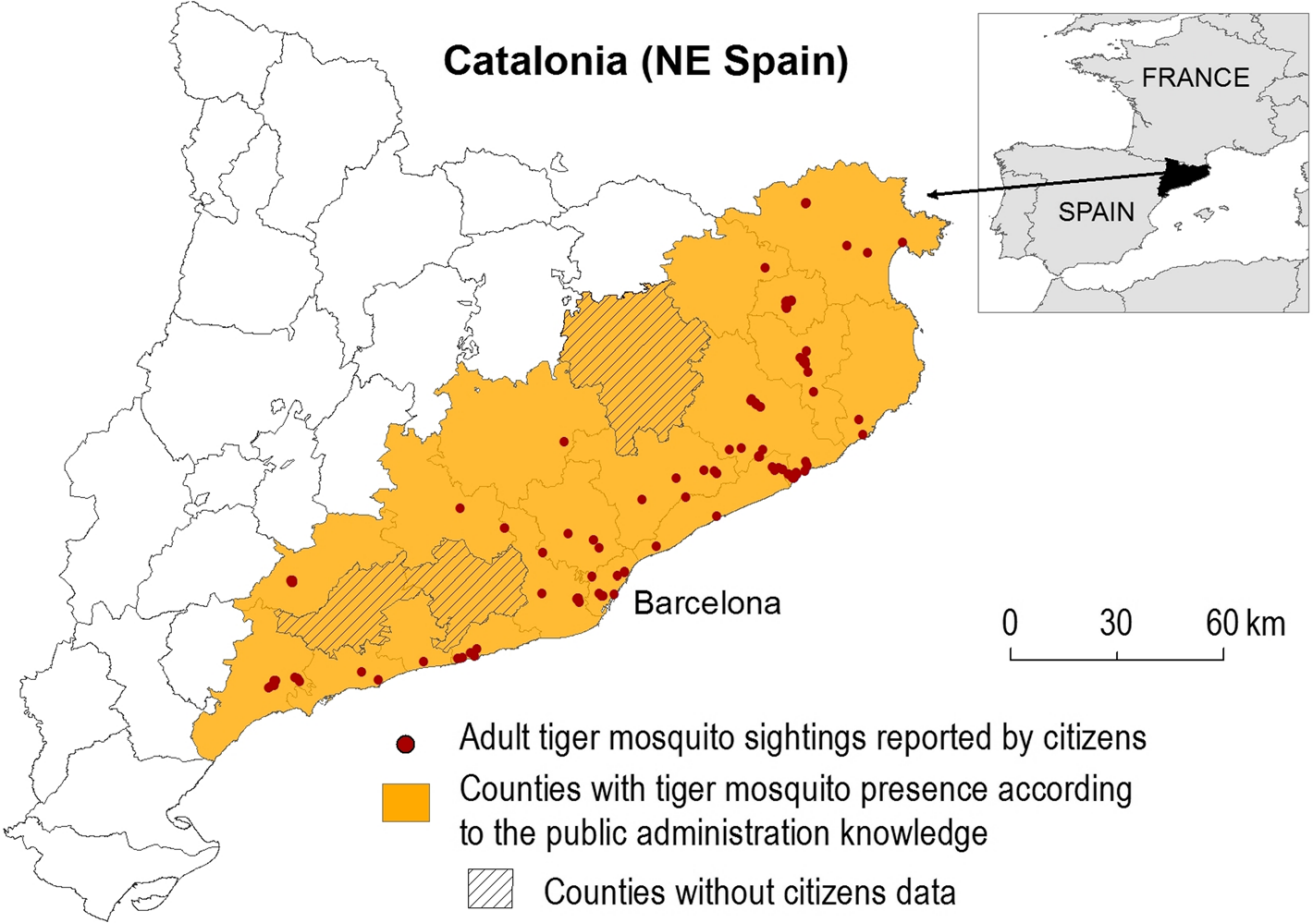
**Comparison of adult tiger mosquito sightings reported by participants during the Spanish “AtrapaelTigre.com” pilot project and demonstrated presence of** ***Ae. albopictus*** **in Catalonia at county scale (as obtained from mosquito control services and public administration personal communication).** Catalonia Basemap: Institut Cartogràfic de Catalunya^©^.

Interestingly enough, however, the almost 150 sightings of adult tiger mosquitoes reported by volunteers, approximately mirrored the known distribution of *Ae. albopictus* in Catalonia at the county scale (Figure 4). It was also demonstrated that specimens sent by post could be used for further genetic analysis, including microsatellites.

Thanks to this participatory process, the tiger mosquito was again very present in the media, helping spread the word on individual actions that citizens can apply at their households to prevent breeding and spread.

Lessons learnt are shaping the continuation of the project, which is now open to all citizens and includes new citizen engagement elements. The app and server components are licensed as free and open source software. The app is now available in three languages (Catalan, Spanish, English), and technological barriers are mostly eliminated.

The project tries to ensure that the privacy of participants is fully protected in all stages. The information collected is not, inherently, of a personal or private nature (e.g. locations, photographs and notes of mosquitoes and breeding sites), which allows working towards a more open approach. In this sense, users are informed during registration that any information they submit through the app may be made public. In fact, avoiding the collection of personal data facilitates a direct return in a real time web map and sharing data with citizens.

Up to now, the app has been downloaded more than 6,000 times and citizens have contributed sending more than 1,400 geolocations of possible tiger mosquito sightings. Between June and September 2014, approximately 150 potential breeding sites were reported, accompanied by some 700 pictures. Data is being validated using novel techniques such as crowd-crafting tasks for pictures, and dynamic and geo-located missions (e.g. encouraging citizens to complement data reports with pictures). Missions permit refining the information sent by citizens in time and space according to specific socio-environmental or scientific interests. This increase in participation and data availability, as compared to the 2013 pilot, calls for a long-term investment and high flexibility.

### France: iMoustique*®*

To implement investigations targeting invasive mosquito species, in particular *Ae. albopictus*, and to prevent the (re-)emergence of mosquito-borne diseases, French public mosquito control agencies have been organized in a network since 1998 [54-56]. Mosquito monitoring initially concentrated on the premises of used tyre trade companies, due to the major mode of international transportation and introduction [57], resulting in the first finding of *Ae. albopictus* in France in 1999 [58]. In 2004, *Ae. albopictus* had finally established in urban areas in southern France, close to the Italian border [5].

**Figure 5:**
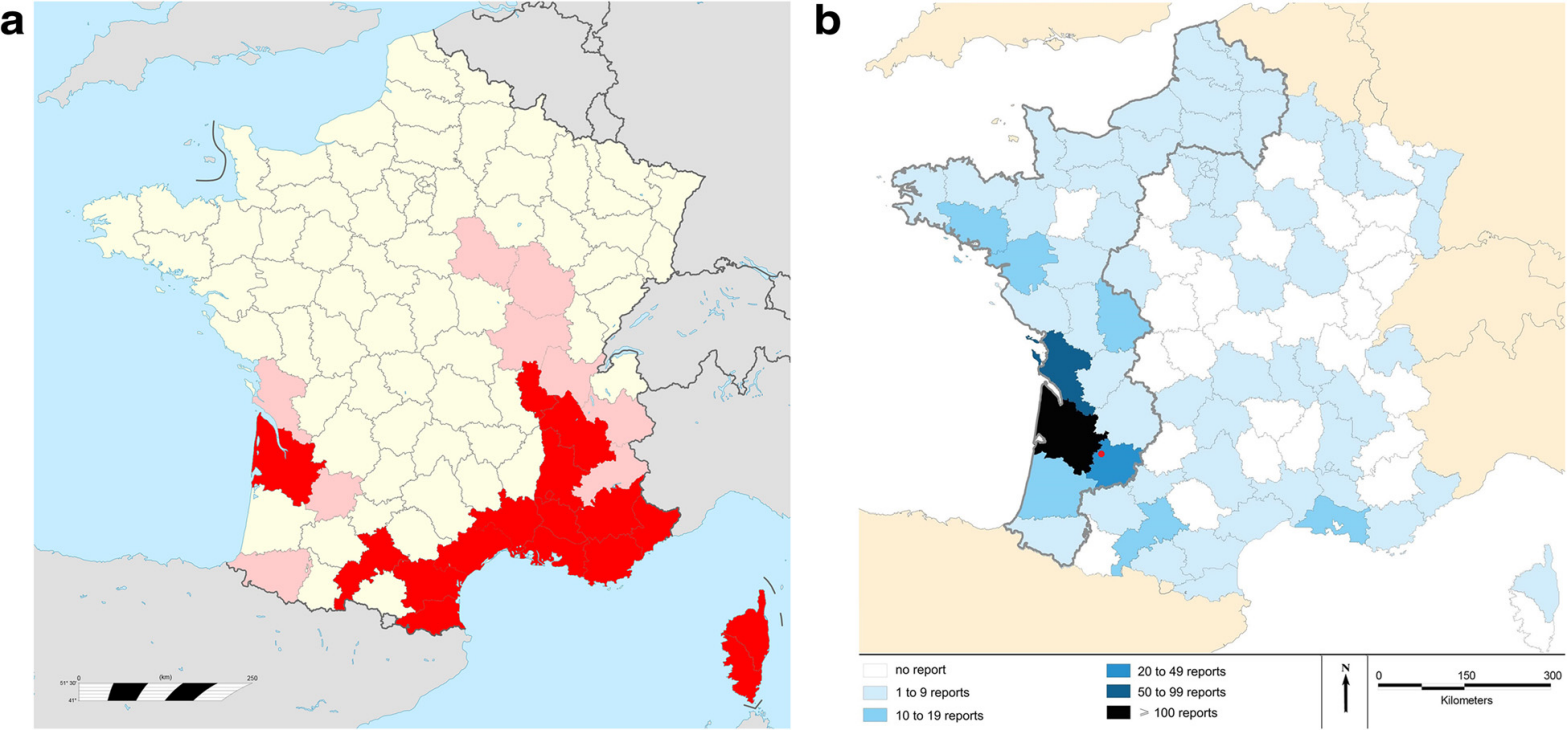
***Aedes albopictus*** **as detected in France and mosquito reports to EID Atlantique. a.** *Aedes albopictus* in France until 2013 (dark red: established populations, light red: occasional detection). **b.** Geographical distribution of mosquitoes reported to EID Atlantique in 2013 (area actively surveyed by EID Atlantique for *Ae. albopictus* is encircled in bold; red dot: location of Beaupuy). France Basemap: GEOFLA^®^IGN.

Considering the presence of *Ae. albopictus* in the metropolitan territory, the French Ministry of Health set up a plan against the spreading of chikungunya and dengue fevers in 2006 including monitoring and controlling invasive mosquitoes [59]. Until 2010, surveillance of invasive mosquito species was essentially based on a network of traditional ovitraps located along the motorways coming from colonized areas in France and close to the borders with countries where *Ae. albopictus* was present. In 2013, *Ae. albopictus* populations were demonstrated to be established in 18 departments (counties) located in southern France (regions of Provence-Alpes-Cotes d’Azur, Languedoc-Roussillon, Rhône-Alpes, Midi-Pyrénées, Aquitaine and Island of Corsica). Occasional detection of the species was made in nine further departments (Figure 5a).

On behalf of the French Ministry of Health, EID Atlantique has been put in charge of surveying 28 departments of the West Atlantic coast from the Spanish border to the Belgian one. For financial reasons, it was clear that it was impossible to monitor a network of traps in close clusters all over this territory. So in 2010, EID Atlantique began to address the community to report the presence of any kind of mosquito by diffusing, through its website, a reporting procedure asking to send samples of mosquitoes by post or pictures by email. In 2011, an information leaflet on mosquitoes was circulated and a contact form on the EID Atlantique website created. The feedback was disappointing: less than 50 reports were received over those two years.

In 2012, the reporting procedure was complemented with a quick response code (flash code) allowing people with a mobile phone to connect directly to a web reporting form. For that year, around 400 reports were received from 52 departments (approximately half of France). For the first time, some reports (6%) referred to *Ae. albopictus*. These could subsequently be confirmed by EID Atlantique staff in three municipalities in the department of Lot-et-Garonne around the city of Marmande through traditional mosquito trapping and collection techniques. However, only 31% of the reports were on mosquitoes while 69% dealt with other insects.

Based on the observation that most of the reports were web-based, the idea emerged to use novel technologies to intensify reporting and enlarge surveillance. In 2013, EID Atlantique developed the first mobile application on mosquitoes (iMoustique®) which allows users to directly transmit a mosquito picture from a cell phone device to a database. As modern mobile devices are equipped with a camera and GPS, users are able to take pictures anytime and anywhere and geo-reference their findings. All mosquito reports are automatically entered into a database including the date of receipt of the report, the reporting mode (web-site form, mail contact, post mail or iMoustique® report), the name of the reporter, her/his department and city, email address and phone number. Each reporter receives a response adapted to the report, be this on an insect other than a mosquito, a native mosquito or an exotic one.

To help people recognize mosquitoes, iMoustique® provides a simple three step determination key. First people have to assess the size of the collected insect in relation to a 20 Euro-cents coin. If the sample is larger, it is not a mosquito; if it is smaller, the participant has to decide whether the insect body is slender and has got long legs. If the answer is no, the insect is not a mosquito but another insect. The last question asks if the insect bears mouthparts looking like a needle. If so, it could be a mosquito and the user can make a report.

The iMoustique® app is an effective way to share information on mosquito presence. Different menus are available to teach people how to be a good reporter. Answers are given to the most common questions on mosquitoes and some information is provided on native mosquitoes described in western France.

In 2013, a total of 602 reports were received (50% more than in 2012), some of which permitted the confirmation of the establishment of *Ae. albopictus* in the town of Beaupuy close to the city of Marmande (department of Lot-et-Garonne; Figure 5b). Two hundred and five (34%) reports were recorded via iMoustique® while the other sources were the contact form from the web site (35%), phone calls (11%), emails (9%) and mails (9%).

iMoustique® was also a way to enlarge the surveyed territory: the mosquito reports in 2013 were submitted from 67 counties in France, 28% more than in 2012 (Figure 5b). But the main result was that close to 90% of the reports (75% more than in 2012) were actually referring to culicids (87% consisting of 15 native species, 3% *Ae. albopictus*). Seventy-seven per cent of the reports were received between June and August, with a maximum in July (249 reports = 41%), owing to the weather conditions during spring, which were particularly supportive to mosquito development in southwestern France.

Considering the current spread of *Ae. albopictus* in France, entomological monitoring by traditional trapping methods reached some limits. The objective of iMoustique® was therefore to facilitate community participation. This novel tool has shown to be able to early detect exotic mosquitoes and to contribute to a better knowledge of native species. The educational approach strengthens the national mosquito monitoring network and enhances the vector risk awareness in an integrated strategy preventing mosquito-borne diseases. iMoustique® is one way to inform and sensitize people to contribute to maintaining public health.

### Portugal: MosquitoWEB

“MosquitoWEB” was launched in April 2014 and is coordinated by the ‘Instituto de Higiene e Medicina Tropical (IHMT), Universidade Nova de Lisboa’. It aims at providing a cost-effective nation-wide mosquito monitoring programme that will complement other on-going surveillance projects in Portugal, such as REVIVE [60], by including the broad community.

The mainstay of the programme is a website (www.mosquitoweb.pt; http://mosquitoweb.ihmt.unl.pt) dedicated to the project. Participants are enrolled in the programme by accessing a webtool directly via their computers or indirectly by telephone contact with IHMT. On the website, the public is informed about the project objectives, and a two-minute video is presented that exemplifies how to capture and submit specimens to IHMT. By filing an iconographic questionnaire and providing a contact point, an automatic email reply is generated. This acknowledges the citizen’s participation and delivers a tag with a serial number and a postal license. This tag allows the mailing of the specimens without expenses to the participant. The iconographic questionnaire provides basic information on the insects’ collection locality.

After morphological and/or molecular identification of the specimen(s) a new message is sent to the participant with the identification of the insect, a brief description of its biology and advice regarding individual protection against mosquito bites.

Besides the detection of newly arrived species, “MosquitoWEB” also provides updated information regarding distribution areas, seasonality patterns and nuisance activity of native mosquito species.

To raise public awareness to “MosquitoWEB” and to enhance community compliance to the project, a media-based promotion plan is to be run between May and July of each year. In the first year, press releases, TV, radio and newspaper interviews were the main focus of the promotion. A roadmap for the presentation of “MosquitoWEB” to the community will also be implemented with municipal authorities targeting civic sectors related to education, health and tourism.

## Discussion

Being fundamental to evidence-based science, active data collection, e.g. by carrying out experimental studies and field work, can be laborious, time-consuming and cost-intensive cf. [61]. As human and financial resources diminish while data requirement increases with scientific progress, more and more scientists make use of passive ways of data collection.

There are two principal approaches of passive data collection in biology, representing various qualities: in case biological, ecological and morphological data on species are needed, data deposited in historical collections may be used. Data may then be extracted from databases (built up by experts or lay people or both), literature collections and voucher specimen/museum collections. In contrast, occurrence and distribution data are usually required to be up-to-date, e.g. when they are to be used for risk assessments, and, for statistical reasons, to be comprehensive. In this case, data collection may be substantially supported by the community.

Approaches to mosquito data collection by passive surveillance, including community-based (so called citizen science) projects, have emerged in various European countries over the past few years. Six of them are presented with regard to organisation, technical challenges and major scientific outcomes. From these projects, the following conclusions can be drawn:

i. Passive surveillance proves to be cost/benefit-efficient and generally results in a large number of data; these quantities, especially the number of sampling locations, are not possible to achieve in standard trapping projects with comparable effort. Thus, passive surveillance may substantially reduce the costs associated with field work incurring in active surveillance programmes. Resources can therefore be concentrated on active surveillance at hotspots, in parallel to, and/or as a consequence of, passive surveillance.
ii. The quality of passive surveillance data is generally good although a considerable portion of arthropods submitted by the public within the framework of citizen science projects are insects other than mosquitoes. Experiences from The Netherlands and France show that communication strategies that focus on distinguishing mosquitoes from other insects will increase the relative proportion of culicids in the overall sample.
iii. Due to the large numbers of locations covered and of mosquitoes reported/submitted by the general public, developments not necessarily foreseeable, such as species establishment, spread, mass development and nuisance, can be detected. It should be stressed that active surveillance must follow on from passive surveillance should certain reports/findings attract attention. Passive surveillance data are generally only appropriate to provide presence information and need confirmation. When additional information is needed, active surveillance must be put in place.
iv. In citizen science, active communication of the project and its results in a transparent way is crucial to stimulate media and public attention. Also, recruitment, instruction and motivation of volunteers depend on the media coverage of the project and on direct eye-to-eye level communication between scientists and participants. Participants should ideally receive feedback on their reports/findings and the relevance of these in the context of public health. It is also important to communicate whether control measures are considered necessary and how the public could support them.
v. Citizen science projects are appropriate to raise awareness and improve knowledge amongst citizens on entomological issues, invasive species and associated public health problems.

Although having started very recently, the various passive mosquito surveillance projects running in EU member states have already collected data in a quantity that a scientist is not able to generate by him-/herself alone. In addition to the huge body of data, unexpected and surprising results have been produced such as the detection and spread of *Ae. albopictus* and *Oc. j. japonicus* populations and the emergence of *Cx. modestus* in some European areas. Actively, these findings would have probably only been obtained with much more investment or over much longer periods of time. A short time period to react to a new situation, however, may be crucial when it comes to control measures and the attempt to eliminate an introduced mosquito species [e.g. 62].

In contrast to citizen science projects that rely on notifications of observations only, the participants of most of the presented projects were given the opportunity to submit mosquito material for further scientific analysis. In these cases, species identification was eventually performed by the scientists themselves thereby guaranteeing a high scientific quality of the data collected. With regard to data verification, citizen science projects have clear advantages over data acquisition from databases or literature, which just have to be believed in being correct.

All described projects are based on elaborate websites and communication both with the participants and the media. Publicity is of major importance, and the special challenge of community-based projects is to appropriately address the public and to keep their interest in participating alive. The media are the most important tools to address citizens and to call their attention to the projects. For the participants there is no remuneration for reporting, collecting and sending mosquitoes; some projects do not even cover the postage when mosquitoes are submitted. Besides education and occasional advice, the compensation for participating is primarily mental.

Fortunately for the scientists, mosquitoes are not just abstract research objects. Although the common interest in them might be smaller than in other, putatively more beautiful and larger animals such as butterflies or birds, the demand to learn about them is wide-spread since almost everybody has at least once been bothered by mosquitoes in their lives and must expect to have negative encounters again in the future. The project participants would therefore like to become informed and educated on mosquitoes and the health risks they pose. Some are really interested in science and wish to contribute to research, provided the tasks given to them are clearly outlined, comprehensible and not too sophisticated. Others might just want to identify nuisance pests and collect information on possible protection and/or control measures in their private domains.

A perspicuous response to the citizens’ reports and submissions will keep them tied to the project and will attract new contributors. Thus, dissemination of information on mosquitoes, an appealing and informative website, communication with the public in general and with participants in particular, as well as identification of the topic with a certain project or a certain research group are crucial to the success of such projects [63].

## Conclusions

As indicated, passive mosquito surveillance supplements, but does not replace, active surveillance. While passive surveillance can reliably provide presence data over a large geographic region, active surveillance is usually more targeted in its aims and appropriate to collect data of a more specific quality, such as abundances, seasonal activities, breeding site and other ecological characteristics, and provide samples for pathogen screening. Thus, passive surveillance may function as a background alert system for triggering active surveillance when necessary and may design more cost-intensive active surveillance activities in a focused way.

While active surveillance is increasingly being standardized on a European level [61,64], there is no international coordination (methods, databases, communication techniques) in passive surveillance so far, although passive surveillance *per se* has lately been promoted by the ECDC [64]. The relatively few approaches to passive surveillance initiated in Europe vary considerably, depending on the specific research question addressed, and cooperation between countries is therefore presently only by exchange of experiences. Discussions on how to use resources across borders and achieve synergies have started, though, and the experiences made within the present various national passive surveillance projects will provide valuable baselines for future collaboration on an international level. Linking and streamlining such initiatives between EU countries and beyond will eventually provide a much better picture of the occurrence, distribution and spread of both native and invasive mosquito species, including potential vectors of disease agents.

## Abbreviations

MRS: Mosquito Recording Scheme
PHE: Public Health England
HPA: Health Protection Agency
CIEH: Chartered Institute of Environmental Health
EPO: environmental protection officer
ICREA: Institució Catalana de Recerca i Estudis Avançats
CEAB-CSIS: Centre d’Estudis Avançats de Blanes - Consejo Superior de Investigaciones Científicas
FECYT: Fundación Española para la Ciencia y la Tecnología
App: Application
GPS: Global positioning system
EID Atlantique: Établissement Interdépartemental pour la Démoustication du littoral Atlantique
IHMT: Instituto de Higiene e Medicina Tropical
REVIVE: Rede de Vigilância de Vetores
ECDC: European Centre for Disease Prevention and Control
